# Optimal Cluster Head Selection in WSN with Convolutional Neural Network-Based Energy Level Prediction

**DOI:** 10.3390/s22249921

**Published:** 2022-12-16

**Authors:** Sasikumar Gurumoorthy, Parimella Subhash, Rocio Pérez de Prado, Marcin Wozniak

**Affiliations:** 1 Department of Computer Science and Engineering, Jerusalem College of Engineering, Chennai 600100, India; 2 Department of Computer Science and Engineering, VNR Vignana Jyothi Institute of Engineering and Technology (VNRVJIET), Hyderabad 500090, India; 3 Telecommunication Engineering Department, University of Jaén, 23700 Linares, Spain; 4 Faculty of Applied Mathematics, Silesian University of Technology, 44-100 Gliwice, Poland

**Keywords:** WSN, improved DCNN, trust evaluation, RSSI, security

## Abstract

Currently, analysts in a variety of nations have developed various WSN clustering protocols. The major characteristic is the Low Energy Adaptive Clustering Hierarchy (LEACH), which attained the objective of energy balance by sporadically varying the Cluster Heads (CHs) in the region. Nevertheless, because it implements an arbitrary number system, the appropriateness of CH is complete with suspicions. In this paper, an optimal cluster head selection (CHS) model is developed regarding secure and energy-aware routing in the Wireless Sensor Network (WSN). Here, optimal CH is preferred based on distance, energy, security (risk probability), delay, trust evaluation (direct and indirect trust), and Received Signal Strength Indicator (RSSI). Here, the energy level is predicted using an improved Deep Convolutional Neural Network (DCNN). To choose the finest CH in WSN, Bald Eagle Assisted SSA (BEA-SSA) is employed in this work. Finally, the results authenticate the effectiveness of BEA-SSA linked to trust, RSSI, security, etc. The Packet Delivery Ratio (PDR) for 100 nodes is 0.98 at 500 rounds, which is high when compared to Grey Wolf Optimization (GWO), Multi-Objective Fractional Particle Lion Algorithm (MOFPL), Sparrow Search Algorithm (SSA), Bald Eagle Search optimization (BES), Rider Optimization (ROA), Hunger Games Search (HGS), Shark Smell Optimization (SSO), Rider-Cat Swarm Optimization (RCSO), and Firefly Cyclic Randomization (FCR) methods.

## 1. Introduction

A Wireless Sensor Network (WSN) contains wide-ranging sensors associated with a wireless medium [1,2,3]. The WSN is employed in an assortment of appliances such as meteorological data compilation, climate monitoring, field surveillance, transport, and healthcare [4,5,6]. On the other hand, the SNs in WSN do not include any rechargeable storage devices and the capability of rechargeable batteries. In this way, it is difficult to sustain any system with capable power utilization. 

Clustering is a major method for making data transmission more effective concerning energy and power utilization. This model split the Sensor Nodes (SNs) into assorted clusters [7,8,9,10]. All clusters in the network have distinguishing CH, which is liable to communicate info to other SN in its cluster. In such a scenario, the chief task is to decide on optimal CH under different constraints, such as lower energy consumption, delay, etc. [11,12,13]. As a result, to form clusters via the data aggregation and fusion systems, there is EE in the network as the quantity of data conveyed to the Base Station (BS) is lessened significantly [14,15,16]. The ageing population, safety, security, healthcare, and other social concerns that advanced nations face are significant issues. The concept of “smart cities” makes use of IoT technology to enhance social infrastructure and create a creative remedy for the aforementioned problems. The researcher is encouraged to employ ubiquitous intelligence by the significant advancements in wireless networks, mobile ad hoc networks, 5G, and fiber optic telecommunication [17]. Nowadays, creating an effective WSN is a key study field. A wireless sensor network (WSN) is a collection of parts that transmits data wirelessly from source to destination. Because of the locations where they are deployed, Wireless Sensor Networks (WSN) always require energy. Furthermore, although energy consumption reduction is not the only cause for clustering, in certain survey reports it has been studied from that perspective. The majority of the clustering publications examine and contrast clustering approaches and their effectiveness but neglect to analyze the strategies’ goals. To the best of our knowledge, no survey research has examined the properties of WSN networks that existing clustering algorithms enable, such as heterogeneity and mobility.

Thus, the cluster-oriented approaches were also affianced in enabling the development of the life span of the network [18]. The most generally employed methods include Fuzzy C-Means (FCM) and Low Energy Adaptive Clustering Hierarchy (LEACH). Furthermore, LEACH was a cluster-oriented method, which operates in a dispersed way that prefers CH, depending on the determined likelihood. Therefore, improving the energy efficiency of the WSN is important because higher energy usage reduces the network’s lifetime. Clustering is an effective tactic for lengthening the network’s lifespan and reducing the amount of transmission energy used. With clustering, packet collision is eliminated, throughput is increased, and network scalability is prolonged. Clustering also decreases overall energy consumption. A set of central cluster-oriented methods have been launched so far, depending on meta-heuristic schemes. Definite wide-ranging schemes are Particle Swarm Optimization (PSO), the Harmony Search Algorithm (HSA), and so on. In addition, the challenging factors in modeling the routing schemes are the life span, QoS, and EE of the network. Mobile sinks are employed in the traditional model because of their high throughput and lower energy use, but information loss is the main disadvantage of utilizing a mobile sink [19]. When transmitting data packets from the source node to the destination node using the suggested architecture, information loss is prevented, and by employing the static node, energy consumption is also decreased. Because of its mobility, a cluster head used in combination with a mobile sink causes a larger loss of data during transmission. Due to its immobility, the cluster head has a lower possibility of losing data when utilized with a static sink. Using the suggested model results in a decrease in energy use and an increase in lifetime. The proposed protocol boosts the cluster’s, group’s, and network’s efficiency in terms of overheads, energy use, and throughput dependability. The lifespan of the network, group members, and cluster head/core are all improved by this efficiency and reliability. 

### 1.1. Main Contribution

- A CHS model for safe and energy-conscious routing in WSNs has been created. Distance, energy, security (risk likelihood), latency, appraisal of trust (direct and indirect trust), and received signal strength indicators all favor CHS (RSSI).
- The proposed method predicts energy level using enhanced Deep Convolutional Neural Network (DCNN) to select the best CH in WSN. The BEA-SSA is used to select the best CH in the WSN. High RSSI, PDR, and residual energy are presented by the Bald Eagle Assisted SSA (BEA-SSA) model. The enhanced energy evaluation and prediction utilizing DCNN and the implemented BEA-SSA-based CHS are responsible for these improvements.
- The proposed cluster head selection process aids LEACH to preserve the network lifetime. The literature includes research that similarly aids LEACH. This paper presents an advanced approach that supports LEACH more effectively.

### 1.2. Organization 

The paper is organized as follows: Previous work is reviewed in Section 2. The network model and parameters for the best CH election are briefly discussed in Section 3 and Section 4. The proposed BEA-SSA model is shown in Section 5. The results and conclusion are further illustrated in Section 6 and Section 7.

## 2. Literature Review

In the related works section, 13 papers are considered. The methods, advantages, and disadvantages of the existing methods are provided.

### Related Works

The firefly contribution using Firefly Cyclic Randomization (FCR) for Cluster Head Selection (CHS) in WSN was proposed by Amit et al. in 2022 [20]. Based on three distribution functions, the method finds the randomly generated solution. Additionally, by changing the random vectors of GWO, the study is based on the second method, Firefly Cyclic Grey Wolf Optimization (FCGWO). The study is carried out and described in terms of network longevity, energy efficiency, and live nodes for the three distributions.

In 2021, Shyjith et al. [21] suggested the hybrid optimization technique for CH selection. The preparation phase, transmission phase, and measuring phase are the three steps that make up the proposed CH selection. Initialization of the network’s energy and node mobility occurs initially. Here, multi-objective constraints that take into account delay, energy, and distance are used to select the threshold and CH. Data transfer from CHs to BS starts after the CHs have been identified. The residual energy generated by the nodes is finally updated during the measurement phase.

In 2019, Reeta et al. [22] designed a multi-objective system that depended on delay, traffic rate, energy, cluster density, and distance. Consequently, energy-aware routing was performed using the developed MOFPL. The designed Multi-Objective Fractional Particle Lion Algorithm (MOFPL) system found the best CH from various CH in WSN. The best routing paths were then set up as per deployed multi-objective function. In addition, capable CHS with superior EE was accomplished by the MOFPL method.

In 2020, Augustine et al. [23] offered an improved system for CHS using Taylor Kernel Fuzzy C-Means (KFCM) customized from the KFCM method. The modeled system designated the CH via the acceptability factor, which was reviewed by energy, distance, and trust. In addition, the advantage of the KFCM system was established concerning higher confidence and higher Energy Efficiency (EE).

In 2019, Goswami et al. [24] recognized a cluster formation method using the Firefly (FF) model and the Hierarchical Maximum Likelihood (HML) system in WSN to enhance EE and to lessen the cost-utility. The problems in the FF method were overcome by integrating the Hierarchical Maximum Likelihood HML hypothesis with it. Additionally, the allotment of power in SNs was completed accurately via the maximum likelihood property of HML. Latterly, the outcomes exposed the better cost function of EE using the adopted model.

In 2019, Toor et al. [25] employed a Mobile Energy Aware Cluster Based Multi-Hop (MEACBM) routing scheme for a variety of hierarchical WSNs. As per the novel probability procedure, the CHs were chosen as the finest; mainly, the SNs were elected as the CH, which offered superior energy over other SNs. This method considerably reduced the energy use of SNs to move the info to BS. The outcomes showed improvements over existing models regarding throughput, CH, life span, and dead nodes. 

In 2019, Daneshvaret al. [26] presented a clustering method that chose CHs via GWO. To elect CH, optimization was performed on the basis of leftover energy. In addition, to achieve better EE, the GWO system employed the cluster in a large number of consecutive rounds. This permitted the scheme to collect the energy that was required to improve CHS. Finally, the outcomes recognized that GWO assured a capable network life span.

In 2020, Prachi et al. [27] deployed BOA to decide the best CH from diverse SNs. As a result, the Butterfly Optimization Algorithm (BOA) method aimed to decrease the use of energy and raise the life span of the system. The path amid BS and CH was represented using Ant Colony Optimization (ACO), and it elected supreme routes based on node degree, EE, and distance. Latterly, the advantage of BOA was established for nodes and energy use. 

In 2019, Sanhaji et al. [28] discuss the experience of implementing a suggested neural network-based routing protocol technique. Our method focuses on enhancing clustering performance. An essential component of Wireless Sensor Networks is energy usage (WSNs). In our approach, which is based on the LEACH (Low-Energy Adaptive Clustering Hierarchy) routing protocol and the neural network’s tool, we introduce the criterion of the consumed energy for the process of the election of the Cluster-Head (CH).

In 2022, Krishna et al. [29] created a group with neighboring sensor nodes, in which the clustering process extends the lifespan of WSNs. For handling the data transfer from the other clustering groups to the fundamental sensor nodes, a principal node known as the cluster head is chosen from the group. Additionally, the cluster heads are positioned to send the signal gathered from its sensor nodes to the base stations. Many WSN applications find it difficult to select an ideal node to serve as a cluster head. The neural network algorithm in the proposed work is used to select a cluster head by examining the base station distance, neighbor sensor counts, and residual energy.

In 2021, Kumar et al. [30] provided a method for finding MNs in WSN using the parameters of each SN. In addition to addressing security, this innovation also made data transmission (DT) energy-efficient by selecting a Cluster Head (CH) that was centered on the sensor’s remaining energy. In the Malicious Nodes Detection (MND) phase, the Improved Deep Convolutional Neural Network (IDCNN) locates the MN and isolates it into the malicious list box. The Extended K-Means (EKM) algorithm clusters the Trusted Nodes (TN) in the energy-efficient DT phase, and the t-Distribution-based Satin Bowerbird Optimization (t-DSBO) algorithm chooses a single CH for each cluster that is centered on the residual energy of those nodes. Through the CH, the cluster’s data are sent to the base station (BS).

In 2022, Gul et al. [31] deployed energy-conscious data gathering in robot network clusters. Due to its limited battery life, a UAV collects data from CH robots by visiting a subset of them, whereas a cluster head (CH) robot in each cluster assigns one collaborative job to each cluster member (CM) robot and collects data from the CMs. A UAV’s decision to visit a subset of CHs is based on several considerations, including the battery’s remaining capacity as well as the locations and data quality of each CH robot. CH robots serve as relay nodes for data transmission for non-visited CH robots.

In 2020, Gul et al. [32] incorporated visiting an optimal number of CH robots while minimizing the total energy consumption of the UAV and the cost of data collection from CH robots. The UAV determines which subset of CH robots to visit by taking into account both its battery capacity and the locations of all CH robots. If the UAV is unable to visit every CH robot, the CH robots that the UAV does not visit relay their data to another CH robot, which then forwards it. The cost optimization process includes choosing the transmission channels for transmitting robots. By taking into account the continuous battery capacity for the UAV, our contribution goes beyond the prevailing paradigms in the literature.

Table 1 exhibits the conventional routing models in WSN. Initially, the FCR used in [20] conserved energy in a capable manner with negligible delay; however, computational time was not examined. The RCSO used in [21] offered improved throughput with negligible delay, but cost metrics must be considered. The MOFPL used in [22] offered high network energy with lesser simulation time, but resource management is not analyzed here. In addition, the Taylor KFCM used in [23] provided high throughput with negligible delay; nevertheless, there was no deliberation on real-time experiments. Likewise, the FF-based model used in [24] improved EE efficiently with enhanced EE. However, FF suffered from local search issues. In addition, the MEACBM model used in [25] ensured minimal energy consumption and offered enhanced life span and throughput. Nevertheless, it required deliberation on the scalability of SNs in every cluster. The GWO model used in [26] offered a better life span for the network and balanced energy consumption. Nevertheless, fault tolerance was not considered. In addition, the BOA + ACO model used in [27] provided an additional number of alive nodes with condensed energy utilization. However, there was no consideration of fault tolerance. The LEACH method is deployed in [28]; the delay and loss are reduced, and the major drawback is that it has to consider the time analysis. In the NN model in [29], the advantage is that energy consumption is reduced and the throughput is high. The experimental analysis should be considered as the major drawback of the system. The IDCNN method is employed in [30]; the main advantages are that energy utilization is low and the life span is increased, but the major drawback is that it needs to consider the stability analysis. The cluster head selection is used in [31]; the advantage is that the cost is reduced, and the drawback is that it needs to consider the scalability analysis. Finally, cluster head selection is deployed in [32]; the major benefit is that energy consumption is reduced, and the drawback is that consistency is not maintained.

Algorithm 1 represents the pseudo-code of the proposed model.
**Algorithm 1.** Pseudo-code of the Proposed Model.Input: $\left(x_{i}^{0},y_{i}^{0}\right)$, i=1,2…N Output: Optimal CH Choose the estimated energy of $SW_{i}$ Update the energy by using Equation (7) Update the security by using Equation (14) Update the distance by using Equation (17) Update the delay by using Equation (18) Update the trust by using Equation (19) Update the RSSI by using Equation (20) The objective function is given in Equation (28) Solve for optimal cluster head End

## 3. A Short Portrayal of the WSN Network 

The below section provides a detailed description of the network model, and the overall architecture of the proposed model is also provided.

### Network Model

Spatially dispersed nodes in wireless sensor networks can convey information gleaned from a monitored or controlled field across a wireless channel. Due to the wide range of potential applications, including those for pressure, light, and other variables, WSNs are a significant area of research. The WSN contains varied SNs signified by $Z_{S}$, and an SN acts as an active sensor throughout the broadcast of data amid BS and CH. Habitually, the WSN is related to data sensing, radio communiqué, allotment of the sensor, topology characteristics, and energy utilization. The sensor is distributed arbitrarily in manual mode at every application area. The SNs are linked to form into clusters, wherein the CH is preferred and CH’s count is implied by $Z_{C}$. The SN in the cluster has to encompass a minimal distance from the CH. The whole SNs from the expected area gather correlated data and communicate it to the CH. The linked CH transmits the information to the BS. Each sink node’s transmission pattern is based on a uniform distribution and distributes data over the maximum radio range possible within the range. This cluster, which takes the shape of a network, is made up of sensor nodes. The phrase designates the relevant cluster head for every cluster. This method of propagating decrease of from the cluster to the BS is collectively referred to as cluster head-based routing. The network model refers to the distance from other nodes to the cluster head as the distance between the cluster head and the base station. For easy transmission, the cluster head is chosen. In the proposed model, the cluster head is chosen based upon the energy, delay, distance, security, trust, and RSSI. To conserve significantly more energy efficiency, power consumption, and energy load balance during each cycle of the sensor nodes, it is interesting to choose a cluster head.

**Figure 1:**
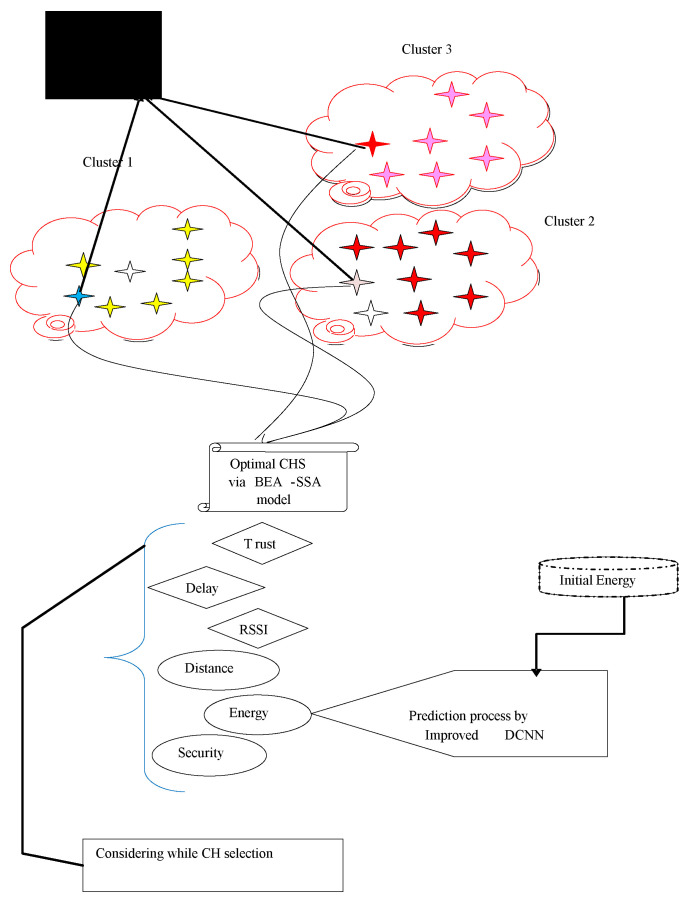
Pictorial representation of the proposed method. Different color star denotes three cluster groups, where cluster head is selected and connected with the base station.

The best way to identify the CH is to pick the node that uses the least amount of energy, allowing the CH to send out more data packets. The main factors used to identify the CH are energy or distance. Furthermore, security is regarded as a crucial component because it ensures protected data transmission. The pictorial representation of the BEA-SSA model is shown in Figure 1. Wherein, three cluster groups (Cluster 1, Cluster 2 and Cluster 3) are considered, from each group, CH is selected and connected with the base station. Here, the selection of the CH depends upon the following constraints: Energy, Trust, Distance, Security, RSSI, and Delay.

## 4. Parameters for the Optimal Election of CH

The varied constraints used to elect the optimal CH are as below:

- Energy;
- Security;
- Distance;
- Trust (direct and indirect);
- Path quality (reliability);
- Delay.

### 4.1. Energy Model

The chief issue related to WSNs is the exploitation of energy [33]. The re-energizing method is not applied for the WSN battery, and consequently, providing energy is impossible if the battery drops down. In addition, data transmitted to the BS from whole SNs is capably made via supplementary resources. Energy exploitation is very important to transmit data. The network needs supplementary energy owing to varied functions that comprise transmission, reception, aggregation, and sensing. Conventionally, the required energy for the overall broadcast of data is implied by Equation (1); however, as per the proposed energy model, the required energy for an overall broadcast of data is implied by Equation (2), where $E^{i}$ denotes initial energy. As per the specified modules, the electronic energy is implied as $E_{el}$, in which $E_{ea}$ denotes the energy for data time aggregation. $E_{el}$ is implied by Equation (4). $E_{TX}(Z:di)$ indicates the whole deployed energy needed to communicate Z bytes of packets at di distance. For a good system, energy must be high.

The receiver energy, $E_{RX}$, required to obtain Z bytes of packets at di is shown in Equation (5), which indicates the energy necessary for amplification, $E_{am}$.

(1) $$E_{TX}(Z:di)=E\left\{ \begin{array}{l} E_{el}\times Z+E_{rs}\times Z\times di^{2},if\;di<di_{0}\\ E_{el}\times Z+E_{pw}\times Z\times di^{2},if\;di\geq di_{0} \end{array}\right.$$

(2) $$E_{TX}(Z:di)=E\left\{ \begin{array}{l} E^{i}-E_{el}\times Z+E_{rs}\times Z\times di^{2},if\;di<di_{0}\\ E^{i}-E_{el}\times Z+E_{pw}\times Z\times di^{2},if\;di\geq di_{0} \end{array}\right.$$

(3) $$E_{el}=E_{TX}+E_{ea}$$

(4) $$E_{RX}(Z:di)=E_{el}Z$$

(5) $$E_{am}=E_{fr}di^{2}$$

(6) $$di_{0}=\sqrt{\frac{E_{fr}}{E_{pam}}}$$

In the above equations,

$di_{0}$ represents threshold distance;

$E_{pam}$ represents energy of PA;

$E_{fr}$ represents requisite energy as employing free space approach;

$E_{1}$ represents energy for entire idle state;

$E_{C}$ represents energy cost for whole sense phase. 

The overall energy to transmit data is indicated by Equation (8).

(7) $$E_{total}=E_{TX}+E_{RX}+E_{1}+E_{C}$$

After computing the energy, the energy level prediction can be performed using improved DCNN. Based on this di, the energy of SN can be predicted. For prediction, DCNN is used, which has di as the input to predict the energy. The DCNN detects significant features with no human intermission. However, it requires large amounts of training data. Therefore, an improvisation is performed to enhance the performance of DCNN. DCNN covers three layers, namely, the Pooling layer, the Fully connected layer, and the Convolution layer. All neurons are connected with closer neurons in the preceding layer. At position (r,t) in the lth layer of correlated wth feature map, the features were designed as in Equation (8).

(8) $$B_{r,t,w}^{l}=W_{w}^{l}{}^{T}PI_{r,t}^{l}+D_{w}^{l}$$

In Equation (8), $W_{w}^{l}$ represents weight and $D_{w}^{l}$ represents the bias of wth filter linked to the lth layer. At the center location (r,t) of the lth layer, the patch input is indicated as $PI_{r,t}^{l}$. The activation value $\left(act_{r,t,w}^{l}\right)$ related to convolutional features, and $B_{r,t,w}^{l}$ is calculated as in Equation (9).

(9) $$act_{r,t,w}^{l}=act\left(B_{r,t,w}^{l}\right)$$

Pooling layer: For each pooling function, pool (•), related to $act_{m,h,w}^{l}$, the $C_{r,t,w}^{l}$ value is considered, as in Equation (10), in which $NN_{r,t}$ represents neighbor’s close location (r,t). The DCNN loss, PL, is exposed in Equation (11).

(10) $$C_{r,t,w}^{l}=\left(act_{m,h,w}^{l}\right)pool,\forall \left(m.h\right)\in NN_{r,t}$$

(11) $$PL=\frac{1}{wn}\sum_{h=1}^{wn}l\left(\theta ;C^{\left(h\right)},F^{\left(h\right)}\right)$$

As per the improved DCNN, error and MSE are computed as shown in Equations (12) and (13), where $t_{pi}$ and $y_{pi}$ refer to the predicted and actual value of data, i, N refers to total data count, β refers to performance ratio, and W refers to neuron weight.

(12) $$Er=\beta \times \mathrm{MSE}+\left(1-\beta \right)\times \sum_{i}{\left(W_{i}\right)}^{2}$$

(13) MSE=(∏i=1N∏P=1P(tpi−ypi))1N

The common variable associated with $W_{w}^{l}$ and $D_{w}^{l}$ is implied by θ. The output, the labels, and hth input feature is denoted as $F^{\left(h\right)}$, $C^{\left(h\right)}$, and $PI^{\left(h\right)}$. Thus, energy $\left(E^{p}\right)$ is predicted using di.

### 4.2. Security

Security mode: This selects the CH that satisfies the security needs. In Equation (8), $q_{r}$&$q_{s}$ mentions security needs linked with CHS and security rank, in that order. The nodes are signified by CH, if $q_{s}\leq q_{r}$.

Risky mode: Here, an existing CH is elected to achieve optimal CH for capturing each risk. As a result, this mode is called insistent mode during the CHS procedure.

γ-risky mode: The CH enduring the severe risk are chosen in γ-risky mode. Additionally, γ-risk is called $u_{risk}$. Subsequently, γ point out the probability metric with γ=0 and γ=1, much like the other two modes.

The likelihood of security variables is shown in Equation (14). In addition, if the chosen CH achieves the state $q_{s}>q_{r}$, the risk should be less than 50%. If the condition is $0<q_{s}-q_{r}\leq 1$, the selection process would be implemented, and if the state is $1<q_{s}-q_{r}\leq 2$, there will be a delay in the selection process. However, the CHS process would not be completed, and the corresponding function should continue for the state $2<q_{s}-q_{r}\leq 5$. For the security analysis, the risk factor is considered. Therefore, the security must be low.

(14) $$Se=\{\begin{array}{cccc} 0 & if & q_{s}-q_{r}\leq 0 & \\ 1-e^{{}^{\frac{\left(q_{s}-q_{r}\right)}{2}}} & if & 0<q_{s}-q_{r}\leq 1 & \\ 1-e^{{}^{\frac{3(q_{s}-q_{r})}{2}}} & if & 1<q_{s}-q_{r}\leq 2 & \\ 1 & if & 2<q_{s}-q_{r}\leq 5 & \end{array}$$

### 4.3. Distance

The distance of packets communicated to the CH from the usual node and from the CH to the BS is calculated as in Equation (15). For a good system, distance must be low.

(15) $$Di=\frac{Di_{dist}^{\left(m\right)}}{Di_{dist}^{\left(n\right)}}$$

(16) $$\mathrm{where},\;Di_{dist}^{\left(m\right)}=\sum_{q=1}^{d}\sum_{t=1}^{M_{e}}\left\|d_{q}^{norm}-M_{c}^{t}\right\|+\left\|M_{c}^{t}-I_{k}\right\|$$

(17) $$Di_{dist}^{\left(n\right)}=\sum_{q=1}^{d}\sum_{t=1}^{d}\left\|d_{q}^{norm}-d_{t}^{norm}\right\|$$

In Equation (15), m and n denote the counting variables, $d_{q}^{norm}$ and $d_{t}^{norm}$ denote the energy of the pth normal node and the energy of the qth normal node, and $M_{c}^{t}$ is the nodes in the cluster.

### 4.4. Delay

Delay is calculated as in Equation (18), in which, d indicate whole cluster count in the network and $M_{c}^{e}$ implies the associated CH. For a good system, delay must be minimum.

(18) $$De_{del}=\frac{\overset{M_{c}^{e}}{\underset{t=1}{\max\left(M_{c}^{t}\right)}}}{d}$$

### 4.5. Trust

All hops in WSN offer higher trust to assess the trust level amid hops and adjoining hops [34]. There are two factors to evaluate trust: direct and indirect trust, as stated in Equation (19). For a good system, trust must be high.

(19) $$Tr=\left\{Tr^{d}+Tr^{id}\right\}\;$$

Direct trust: calculated as shown in Equation (20), in which, ${\left(Tr^{d}\right)}_{y}^{z}$ denotes direct trust for yth transaction and zth time interval, sm denotes Satisfaction metric, z denotes Time interval, y denotes Transaction, o denotes Estimation hop, and o+1 denotes Hop for assessment.

(20) $${\left(O^{d}\right)}_{y}^{z}\left(o,o+1\right)=sm_{y}^{z}\left(o,o+1\right)$$

In Equation (21), sm is assessed as in Equation (21), wherein $sm_{v}$ denotes satisfaction value of the present transmission, $sm_{y-1}^{z}\left(o,o+1\right)$ denotes y−1 transmission satisfaction value at zth time interval, and η denotes weight.

(21) $$sm_{y}^{z}\left(o,o+1\right)=\eta \times sm_{v}+\left(1-\eta \right)\times sm_{y-1}^{z}\left(o,o+1\right)$$

(22) $$sm_{v}=\left\{ \begin{array}{l} 0;\;if\;\mathrm{transmission}\;\;is\;unsatisfactory\\ 1;\;if\;\mathrm{transmission}\;\;is\;satisfactory\\ \;\in \left(0,1\right);\;else \end{array}\right.\;$$

Indirect trust: Indirect trust of oth hop regarding (o+1)th is assessed as shown in Equation (23), wherein V denotes agent group interacting with o+1, a denotes hop, and $K_{y}^{z}$ denotes feedback creditability.$K_{y}^{z}$ is assessed by Equation (24), in which $L_{y}^{z}$ denotes resemblance. The resemblance amid hops is assessed as in Equation (25), wherein l denotes similarity deviation constant, δ&ϖ denotes punishment and reward factor, and ${ℜ}_{y}^{z}\left(o,o+1\right)$ denotes personalized difference.

(23) $${\left(Tr^{id}\right)}_{y}^{z}\left(o,o+1\right)=\left\{ \begin{array}{l} \frac{\sum_{a\in U-\left\{o\right\}}K_{y}^{z}\left(o,a\right)\times {\left(Tr^{d}\right)}_{y}^{z}\left(a,o+1\right)}{\sum_{a\in U-\left\{o\right\}}K_{y}^{z}\left(o,a\right)};\;if\;\left|V-\left\{o\right\}\right|=0\\ 0;\;if\;\left|v-\left\{o\right\}\right|>0 \end{array}\right.$$

(24) $$K_{y}^{z}\left(o,o+1\right)=\left\{ \begin{array}{l} 1-\frac{\ln\left(L_{y}^{z}\left(o,o+1\right)\right)}{\ln\varphi };\;if\left(L_{y}^{z}\left(o,o+1\right)\right)>\varphi \;\\ 0;\;else \end{array}\right.$$

(25) $$L_{y}^{z}\left(o,o+1\right)=\left\{ \begin{array}{l} L_{y-1}^{z}\left(o,o+1\right)+\frac{1-L_{y-1}^{z}\left(o,o+1\right)}{\varpi }\;;if\;{ℜ}_{y}^{z}\left(o,o+1\right)<l\\ L_{y-1}^{z}\left(o,o+1\right)-\frac{1-L_{y-1}^{z}\left(o,o+1\right)}{\delta };else \end{array}\right.$$

### 4.6. RSSI

RSSI corrupts as the inverse square of distance amid receiver and sender based on Fris [35]. It can be modeled as shown in Equation (26), wherein B refers to distance and is computed as shown in Equation (27). For a good system, RSSI must be high.

(26) RSSI=−36×log(B)−55

(27) $$B=10^{\left(\mathrm{RSSI}+55\right)/-36}$$

### 4.7. Objectives

The intention of the BEA-SSA-based scheme for electing the optimal CH is shown in Equation (28), where di is the distance, $E^{p}$ is the energy, Se is the security, De is the delay, Tr is the trust, and RSSI is the Received Signal Strength Indicator. The objective function is shown below. Our objective function is distance, security, and delay should be minimum, but the remaining parameters of energy, trust, and RSSI must be maximum. Here, for security analysis, the risk factor is considered. Therefore, the risk factor must be minimum.

(28) $$Obj=\min\left[\begin{array}{l} \left(we_{1}\times di\right)+\left(we_{2}\times \left(1-E^{p}\right)\right)+\left(we_{3}\times \left(Se\right)\right)\\ \;+\left(we_{4}\times De\right)+\left(we_{5}\times \left(1-Tr\right)\right)+\left(we_{6}\left(1-\mathrm{RSSI}\right)\right) \end{array}\right]$$

In Equation (13), $we_{1}$–$we_{6}$ are weight factors that ranges from 0 to 1; $we_{1}$ is assigned with 0.2, $we_{2}$ is assigned with 0.3, $we_{3}$ is assigned with 0.1, $we_{4}$ is assigned with 0.2, $we_{5}$ is assigned with 0.1, and $we_{5}$ is assigned with 0.1.

## 5. Developed BEA-SSA for Optimal CHS

Solution encoding: as said earlier, CHs are optimally chosen via the BEA-SSA model. The input solution will be the nodes and the solution length will be 100, from which 10 nodes will be chosen as CH.

### Proposed BEA-SSA Model

To augment the performance of the traditionalist SSA model [36], BES [37] is combined with it to form BEA-SSA. Usually, self-improvement in typical optimization schemes [38,39,40,41,42,43,44] outcome in superior accuracy. The following rules are included in BEA-SSA.

- The producers usually have a significant amount of energy and give foraging locations or guidance to all scavengers. Further, it has the responsibility of locating places with abundant food supplies. The level of energy reserves is determined by an evaluation of the individuals’ fitness values.
- Individual sparrows begin to chirp as alarm messages as they detect the predator. When the alarm value exceeds the safety level, the producers must direct all scavengers to the safe zone.
- Every sparrow becomes a producer as much as it seeks out larger food sources; however, the proportion of scroungers and producers remains constant in the entire population.
- The producers would act as the sparrows with the maximum energy. Numerous starving scavengers are inclined to fly toward different locations in search of food to gain energy.
- Scroungers search for food by following the producer who can supply the healthiest food. Meanwhile, some scavengers might keep a close eye on the producers and fight for food to boost their predation rate.
- When the sparrows present at the group’s edge become aware of the danger, they swiftly move into the safe region to obtain a higher position, but the sparrows in the center of the group arbitrarily walk to be adjacent to others.

The sparrows make use of virtual sparrows for searching for food in the simulated experimentation. As per BEA-SSA, the initialization is done using the chaotic map function via a sinusoidal map, as shown in Equation (29), where $X^{r}\in \left(0,1\right)$.

(29) $$X^{r+1}=bX_{r}^{2}\sin\left(\pi X^{r}\right)$$

Equation (30) is used to represent the position of sparrows.

(30) $$X=\left[\begin{array}{l} X_{1,1\;}\;X_{1,2}\;\ldots \;\ldots \;X_{1,a}\\ X_{2,1\;}\;X_{2,2}\;\ldots \;\ldots \;X_{2,a}\\ .\;.\;.\;.\;.\;\\ \;.\;.\;.\;.\\ \;.\;.\;.\;.\\ X_{s,1\;}\;X_{s,2}\;\ldots \;\ldots \;X_{s,a} \end{array}\right]$$

In Equation (30), s points out the sparrow count and a indicates the optimized variable’s dimension. The fitness of the sparrow is symbolized as in Equation (31), where $F_{X}$ point to the fitness value of the individual.

(31) $$F_{X}=\left[\begin{array}{l} f\left(\left[X_{1,1\;}\;X_{1,2}\;\ldots \;\ldots \;X_{1,a}\right]\right)\\ f\left(\left[X_{2,1\;}\;X_{2,2}\;\ldots \;\ldots \;X_{2,a}\right]\right)\\ \;.\;.\;.\;.\;\\ \;.\;.\;.\;.\;\\ \;.\;.\;.\;.\;\\ f\left(\left[X_{s,1\;}\;X_{s,2}\;\ldots \;\ldots \;X_{s,a}\right]\right) \end{array}\right]$$

The producers with large fitness levels in SSA contain a maximum possibility of attaining food all through the searching phase. The producers are reliable in discovering food and direct the moment of the entire populace; the producers look for foodstuff in more areas compared to scroungers.

The locations of producers are updated based on rules (a) and (b) of SSA, as per Equation (32).

(32) $$X_{c,d}^{r+1}=\left\{ \begin{array}{l} X_{c,d}^{r}.\exp\left(\frac{-c}{\alpha .it_{\max}}\right)\;if\;C_{2}<st\\ X_{c,d}^{r}+P\;.M\;if\;C_{2}\geq st \end{array}\right.$$

In Equation (32), r denotes the present iteration, $it_{\max}$ refers to maximal iteration, α indicates arbitrary integer, st and $C_{2}$ refer to safety threshold and alarm value, P denotes random number with normal distribution, and M Illustrate a matrix of 1 × d with element 1. 

Conversely, the scroungers should pursue rules (d) and (e). As said earlier, various scroungers keep a close eye on producers. Because producers recognize good food, they depart their present position to fight for food. If they reach first, they instantaneously cover the producer’s food, or else they should pursue rule (e). As per BEA-SSA logic, the scrounger’s position is updated using BES, as specified in Equation (33). Here, $X_{mean}$ is computed using the geometric mean of all information from the previous points.

(33) $$X{\left(i\right)}_{new}=X\left(i\right)+Y\left(i\right)\times \left(X\left(i\right)-X\left(i+1\right)\right)Y_{best+}Y\left(i\right)\times ra\left(X\left(i\right)-X_{mean}\right)$$

If c>s/2, it is implied that the cth scrounger with worse fitness value is typically hungry. In addition, the mathematical model as per rules (f) is shown in Equation (34).

(34) $$X_{c,d}^{r+1}=\left\{ \begin{array}{l} X_{best}^{r}+\gamma .\left|X_{c,d}^{r}-X_{best}^{r}\right|\;if\;f_{c}<f_{u}\\ X_{c,d}^{r}+Z.\left(\frac{\left|X_{c,d}^{r}-X_{worst}^{r}\right|}{\left(f_{c}-f_{w}\right)+\varepsilon }\right)\;if\;f_{c}=f_{u} \end{array}\right.$$

In Equation (34), γ indicates the step size control parameter with a variance of 1 and mean value of 0, $R_{best}$ denotes the current global optimal location, Z∈[−1,1] indicates the random number, $f_{c}$ indicates the fitness value of the current sparrow, $f_{w}$ and $f_{u}$ indicate worst fitness values and current global best, correspondingly, and ε indicates small constant for avoiding zero-division-error.

**Figure 2:**
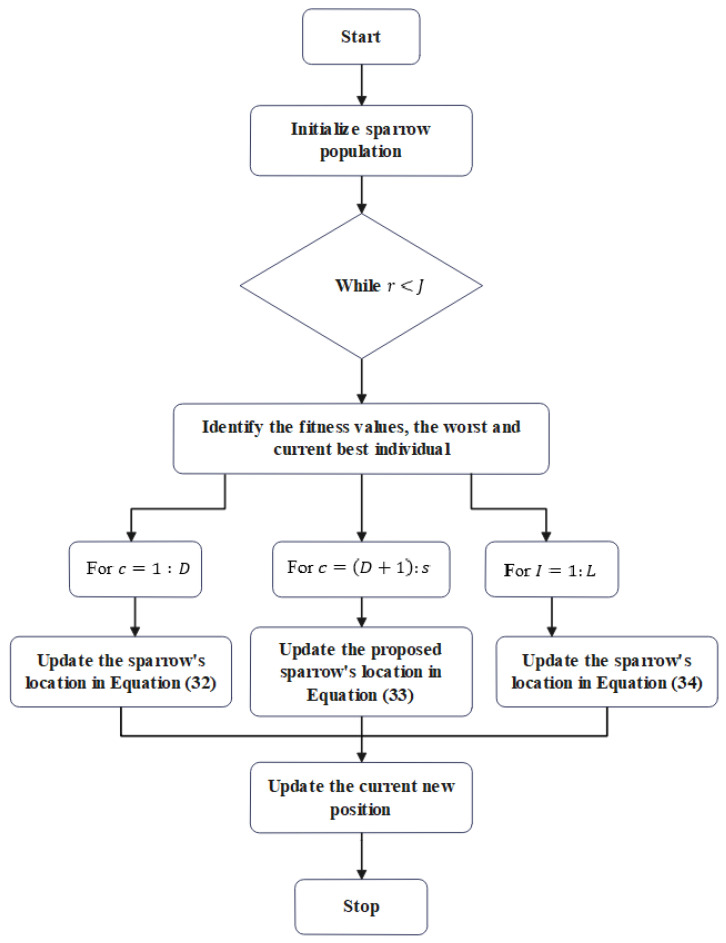
Flowchart of BEA-SSA model.

If $f_{c}>f_{u}$, it indicates that the sparrow is at the edge of the group, then $X_{best}$ denotes the position at the center of the population.$f_{c}=f_{u}$ denotes that the sparrows were at the center of the population, and Z denotes the route of movement of the sparrow. D denotes the count of producers, and L specifies the number of sparrows that perceive the danger. Figure 2 shows the flowchart of the BEA-SSA model.

Table 2 shows the list of symbols.

## 6. Results and Discussion

In the results and discussion section, the simulation parameters are discussed and analyzed regarding the delay, distance, trust, and so on. 

### 6.1. Simulation Procedure

**Figure 3:**
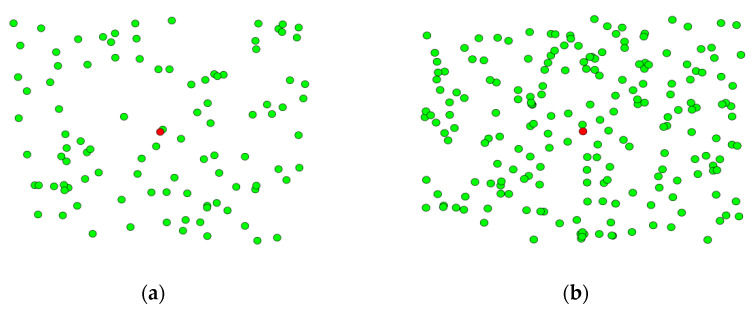
Simulation outcomes using BEA-SSA model for node counts of (**a**) 100 and (**b**) 200.

The employed scheme for CHS in WSNs using BEA-SSA was executed in MATLAB. The constraints utilized for simulation are shown in Table 3. Further, the assessment was performed for two node groups, i.e., 100 nodes and 200 nodes. The study was performed for two variants of nodes concerning delay, PDR, distance, RSSI, security, throughput, alive nodes, trust, and energy. Here, the improvement of delay was confirmed over GWO [26], MOFPL [22], PRO, SSA [36], BES [37], ROA, Hunger Games Search (HGS), SSO, RCSO [21], and FCR [20]. Figure 3 represents the node location of the deployed scheme for 100 and 200 nodes. Here, the centre of the node (sink node) is denoted as red color, where the normal nodes are defined in green color.

### 6.2. Analysis of Delay and Distance

**Figure 4:**
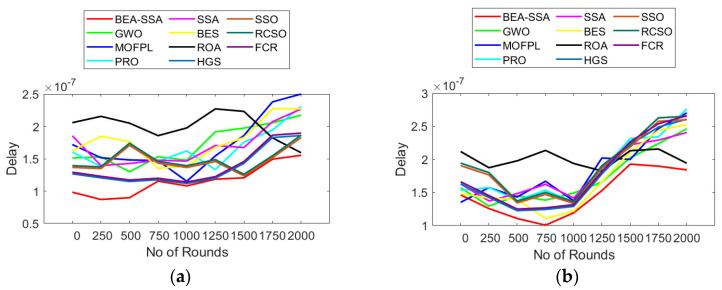
Delay analysis using BEA-SSA compared with other models [20,21,22,26] for node counts of (**a**) 100 and (**b**) 200.

**Figure 5:**
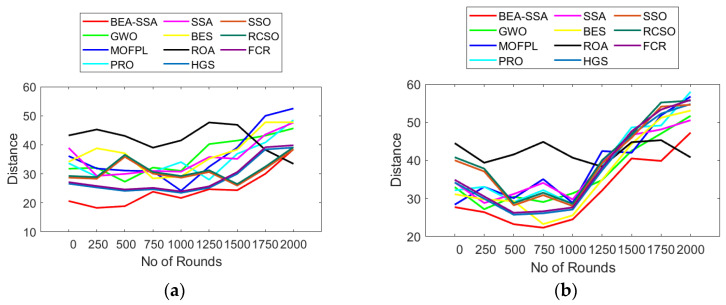
Distance analysis using BEA-SSA compared with other models [20,21,22,26] for node counts of (**a**) 100 and (**b**) 200.

The analysis of delay and distance using the BEA-SSA method compared to GWO [26], MOFPL [22], PRO, SSA [36], BES [37], ROA, HGS, SSO, RCSO [21], and FCR [20] is shown in Figure 4 and Figure 5. The examination was performed for two sets of nodes (100 and 200). The delay for transferring packets must be the smallest for good performance of the system. Likewise, the distance between the CH and the BS should be less for good performance of the system. Here, the delay is symbolized in seconds. In Figure 4, it is noticed that, as the round count rises, the delay also increases. At first, at 0th round, the delay for 100 nodes using BEA-SSA is only 1 × 10^−7^. As the round increases, i.e., at the 2000th round, the delay value for 100 nodes using the BEA-SSA technique is 1.5 × 10^−7^ s. Similarly, as the round count rises, the distance also increases. At the 0th round, the distance for 200 nodes using BEA-SSA is 27. As the round increases, i.e., at the 2000th round, the distance value for 200 nodes using the BEA-SSA technique is 47. Moreover, the BEA-SSA scheme is superior to the compared GWO [26], MOFPL [22], PRO, SSA [36], BES [37], ROA, HGS, SSO, RCSO [21], and FCR [20] models. To choose the best CH in WSN, an improved Deep Convolutional Neural Network (DCNN) predicts the energy level. The RSSI, PDR, and residual energy are all high for the Bald Eagle Assisted SSA (BEA-SSA) model. The WSN uses the BEA-SSA to determine which CH is the best. These developments are attributable to the BEA-SSA-based CHS’s deployment and the improved energy evaluation and prediction using DCNN. This enhancement is due to the improved energy evaluation and prediction using DCNN.

### 6.3. Statistical Performance 

Table 4 and Table 5 show the statistical study for 100 nodes and 200 nodes using the employed BEA-SSA model compared with conventional models (GWO [26], MOFPL [22], PRO, SSA [36], BES [37], ROA, HGS, SSO, RCSO [21], and FCR [20]). The met heuristic schemes are stochastic, and to substantiate their fair evaluation, each model is analyzed a significant number of times to accomplish minimal residual energy. In Table 4, the proposed BEA-SSA technique has achieved minimal residual energy values (−0.00058) for the best case for 200 nodes. Amongst every scheme, conservative PRO has achieved the worst values for every scenario for the GWO [26], MOFPL [22], PRO, SSA [36], BES [37], ROA, HGS, SSO, RCSO [21], and FCR [20] schemes. This is due to the improved energy evaluation and prediction using IDCNN and the adopted BEA-SSA-based CHS.

### 6.4. Analysis of Alive Nodes

**Figure 6:**
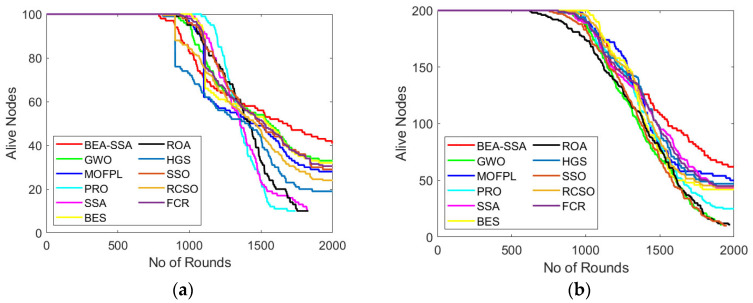
Alive node analysis using BEA-SSA compared with the other models [20,21,22,26] for node counts of (**a**) 100 and (**b**) 200.

The study of the BEA-SSA model concerning alive nodes is shown in Figure 6. The improvement of BEA-SSA over GWO [26], MOFPL [22], PRO, SSA [36], BES [37], ROA, HGS, SSO, RCSO [21], and FCR [20] was established. Here, assessment is carried out for 100 and 200 nodes. The alive node count has to be higher for superior performance. Here, the count of an alive node is lowered with a rise in the count of rounds. In Figure 6a, the alive nodes for BEA-SSA at the 0th round is 100, while at the 2000th round the alive node count for BEA-SSA is 40. Likewise, in Figure 6b, the alive nodes for BEA-SSA at the 0th round is 200, while at the 2000th round the alive node count for BEA-SSA is 65. Thus, for both 100 and 200 nodes, the alive nodes are high, up to 700 rounds; after that, the count of alive nodes starts lessening with the rise in rounds.

### 6.5. Analysis of Residual Energy, RSSI, and PDR

**Figure 7:**
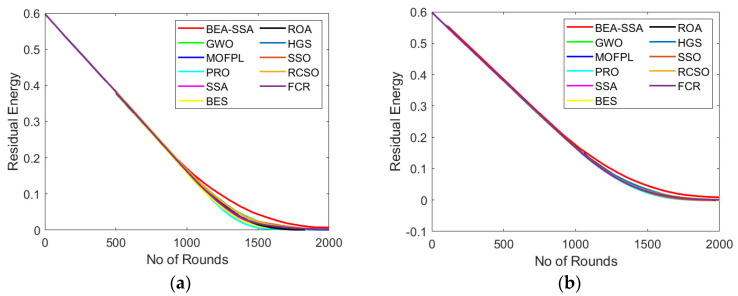
Residual energy analysis using BEA-SSA compared with the other models [20,21,22,26] for node counts of (**a**) 100 and (**b**) 200.

**Figure 8:**
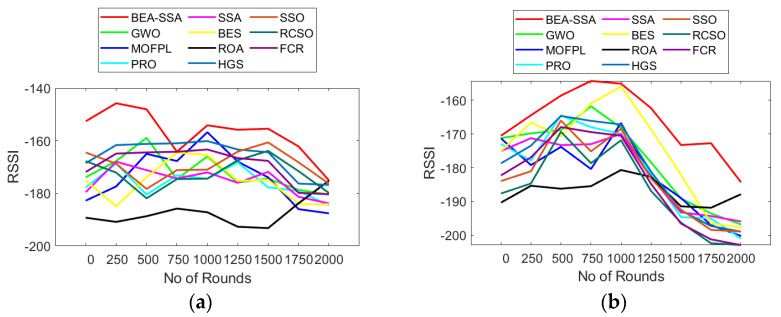
RSSI analysis using BEA-SSA compared with the other models [20,21,22,26] for node counts of (**a**) 100 and (**b**) 200.

**Figure 9:**
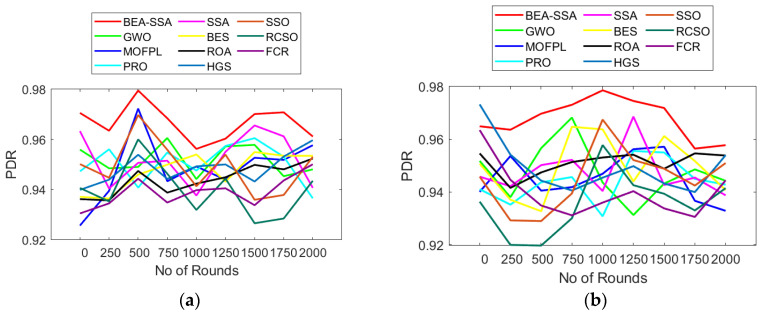
PDR analysis using BEA-SSA compared with the other models [20,21,22,26] for node counts of (**a**) 100 and (**b**) 200.

The RSSI, PDR, and residual energy study using the BEA-SSA method compared with other conservative models, GWO [26], MOFPL [22], PRO, SSA [36], BES [7], ROA, HGS, SSO, RCSO [21], and FCR [20], is portrayed in this section. The more superior the remaining energy, the more superior will be the performance of the system. In Figure 7a, the remaining energy continues to reduce with the rise in rounds. In Figure 7a, the remaining energy for BEA-SSA and conservative models is 0.6 at round 0; however, as the rounds increase, the remaining energy starts deteriorating. Similarly, in Figure 8, the RSSI is high at the 250th round for 100 nodes, while for 200 nodes the RSSI is high at the 750th and 1000th rounds. In Figure 9, the PDR fluctuates for every round. In Figure 9a, the PDR is high at the 500th round for 100 nodes, while for 200 nodes the PDR is high at the 1000th round. However, when compared with GWO [26], MOFPL [22], PRO, SSA [36], BES [37], ROA, HGS, SSO, RCSO [21], and FCR [20], the BEA-SSA model poses high residual energy, high RSSI, and high PDR. An improved Deep Convolutional Neural Network (DCNN) is utilized to choose the best CH in WSN by predicting the energy level. The WSN chooses the best CH using the BEA-SSA. The Bald Eagle Assisted SSA (BEA-SSA) model has high RSSI, PDR, and residual energy. These developments are due to the installation of the BEA-SSA-based CHS and the improved energy evaluation and prediction using DCNN. These enhancements are due to the improved energy evaluation and prediction using DCNN and the adopted BEA-SSA-based CHS.

### 6.6. Convergence Analysis

**Figure 10:**
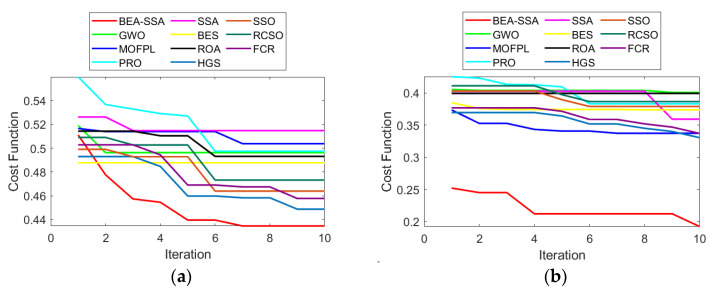
Convergence of BEA-SSA approach compared with the other models [20,21,22,26] for (**a**) 100 and (**b**) 200 nodes.

The convergence of the BEA-SSA technique compared with GWO [26], MOFPL [22], PRO, SSA [36], BES [37], ROA, HGS, SSO, RCSO [21], and FCR [20] for diverse iterations is shown in Figure 10. As needed, the BEA-SSA has achieved the least cost values with an increase in iterations for both nodes. From Figure 10a, from the 7th to the 10th iteration, the cost has reduced to 0.435 for 100 nodes. Likewise, in Figure 10b, from the 4th to the 10th iteration, the cost has reduced to 0.19 for 200 nodes. The other evaluated schemes, GWO [26], MOFPL [22], PRO, SSA [36], BES [37], ROA, HGS, SSO, RCSO [21], and FCR [20], reveal a comparatively elevated cost value. The energy level is predicted using an enhanced Deep Convolutional Neural Network (DCNN), which is used to select the best CH in WSN. The BEA-SSA is used in the WSN to select the best CH. The RSSI, PDR, and residual energy are all high for the Bald Eagle Assisted SSA (BEA-SSA) model. The implementation of the BEA-SSA-based CHS and the enhanced energy evaluation and prediction utilizing DCNN are responsible for these advancements. Thus, with the BEA-SSA-based optimization, improved results are obtained for CHS.

### 6.7. Analysis of Throughput, Trust, and Security

**Figure 11:**
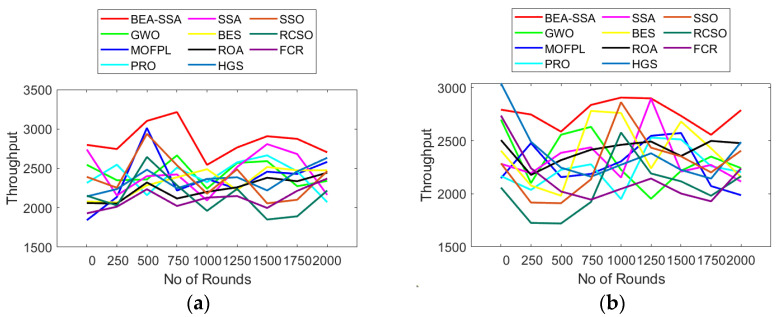
Throughput analysis using BEA-SSA over other models [20,21,22,26] for node count of (**a**) 100 (**b**) 200.

**Figure 12:**
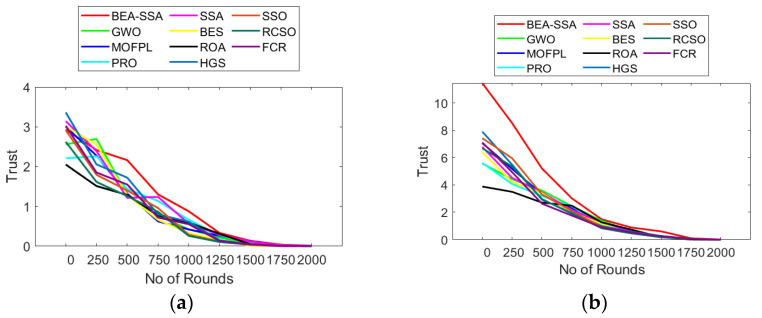
Trust analysis using BEA-SSA over other conventional models [20,21,22,26] for node count of (**a**) 50 (**b**) 100.

**Figure 13:**
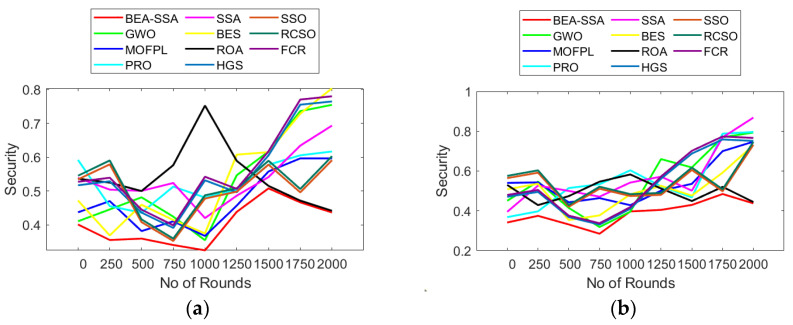
Security analysis using BEA-SSA over other conventional models [20,21,22,26] for node count of (**a**) 50 (**b**) 100.

The analysis of trust, security, and throughput using the BEA-SSA scheme compared with GWO [26], MOFPL [22], PRO, SSA [36], BES [37], ROA, HGS, SSO, RCSO [21], and FCR [20] is shown in Figure 11, Figure 12 and Figure 13. Here, evaluation is performed for 100 and 200 nodes. Throughput is a measure of how many units of information a system can process in a given amount of time. Throughput is usually measured in bits per second (bit/s or bps), and sometimes in data packets per second (p/s or pps) or data packets per time slot. The throughput has to be high as it plays a chief role in transferring data. In Figure 11a, the throughput is high at 750th rounds for 100 nodes, while for 200 nodes the throughput is high at 1000–1250th rounds. In Figure 12, the trust factor is high at the initial rounds, but as the rounds increase, trust values lessen. However, when distinguished over GWO [26], MOFPL [22], PRO, SSA [36], BES [37], ROA, HGS, SSO, RCSO [21], and FCR [20], the BEA-SSA model poses high trust. Likewise, the security in terms of risk is lesser for the BEA-SSA method, while, GWO [26], MOFPL [22], PRO, SSA [36], BES [37], ROA, HGS, SSO, RCSO [21] and FCR [20] shows high-security risk. Chooses the best CH in WSN by predicting energy level using an improved Deep Convolutional Neural Network (DCNN). The BEA-SSA is used to choose the WSN’s top CH. The Bald Eagle Assisted SSA (BEA-SSA) model has high RSSI, PDR, and residual energy. These improvements are attributable to better energy evaluation and prediction using DCNN and the installed BEA-SSA-based CHS. Using an improved Deep Convolutional Neural Network (DCNN), which is used to choose the best CH in WSN, predicts energy level. In the WSN, the best CH is chosen using the BEA-SSA. The Bald Eagle Assisted SSA (BEA-SSA) model has high RSSI, PDR, and remaining energy. These improvements are the result of the BEA-SSA-based CHS being implemented, as well as the improved energy evaluation and prediction using DCNN. This promises the superior performance of the BEA-SSA approach.

### 6.8. Discussion

In the analysis of delay and distance, first, the delay for 100 nodes utilizing BEA-SSA at round zero is just 1 × 10^−7^. At the 2000th round, for example, the delay value for 100 nodes using the BEA-SSA approach is 1.5 × 10^−7^ s. Similarly, as the number of rounds rises, so does the distance. In fact, for the system to operate well, the packet transmission latency must be as minimal as possible. Table 4 shows that in the best-case scenario for 200 nodes, the suggested BEA-SSA approach has significantly reduced residual energy values (−0.00058). It is because BEA-SSA-based CHS was implemented and IDCNN was used to improve energy evaluation and prediction. For both nodes, the BEA-SSA has attained the least cost values as needed with an increase in iteration. The throughput is crucial for data transport; hence it must be high. For 100 nodes, the throughput peaks at the 750th round, while for 200 nodes, it peaks at the 1000th to 1250th rounds. In round 12, the trust factor is high at first, but it decreases as more rounds are played. The BEA-SSA model, however, poses high trust and the security risk is lower for the BEA-SSA approach; however, it is higher for the GWO [26], MOFPL [22], PRO, SSA [36], BES [37], ROA, HGS, SSO, RCSO [21], and FCR [20]. Moreover, the BEA-SSA model posed significant residual energy, high RSSI, and high PDR compared to GWO [26], MOFPL [22], PRO, SSA [36], BES [37], ROA, HGS, SSO, and RCSO [21]. The best CH in WSN is chosen by predicting the energy level using an improved Deep Convolutional Neural Network (DCNN). The BEA-SSA is used to choose the WSN’s top CH. The Bald Eagle Assisted SSA (BEA-SSA) model has high RSSI, PDR, and residual energy. These improvements are attributable to better energy evaluation and prediction using DCNN and the installed BEA-SSA-based CHS.

## 7. Conclusions

This study recommended an optimal CHS model for WSNs’ highly secure and energy-efficient routing. Here, it was decided to use optimal CH in consideration of RSSI, distance, energy, security (risk probability), delay, and trust assessment (direct and indirect trust). Improved DCNN was used in this case to estimate the energy level. BEA-SSA was used to select the best CH in WSN. According to the results, for 100 nodes, the RSSI peaked at round 250, whereas for 200 nodes it peaked at rounds 750 and 1000. Additionally, the PDR reached its peak for 100 nodes at round 500, and for 200 nodes at round 1000. In addition, the PDR was high at the 500th round for 100 nodes, while for 200 nodes the PDR was high at the 1000th round. However, when compared with GWO, MOFPL, PRO, SSA, BES, ROA, HGS, SSO, RCSO, and FCR, the BEA-SSA model posed high residual energy, high RSSI, and high PDR. In the future, fault tolerance should be examined, which will improve the clustering algorithm mechanism.

## Figures and Tables

**Table 1:** Reviews on CHS models in WSN.

Author	Proposed Method	Features	Challenges
Amit al. [20]	FCR	High delay High EE	Computational time needs to be analyzed
Shyjith et al. [21]	RCSO	Less delay Higher throughput	Should consider cost metrics
Reeta and Dinesh [22]	MOFPL	Lesser time Higher network energy	Need to compute cost efficiency
Augustine and Ananth [23]	Taylor KFCM model	High energy and throughput Least delay	No concern about practical experimentation
Goswamiet al. [24]	FF	Negligible cost function Enhanced EE	Endures from local searching issues
Toor and Jain [25]	MEACBM	Minimal energy employment Amplified throughput	No concern about Scalability
Daneshvar et al. [26]	GWO	Lower energy utilization Higher network lifespan	No concern about Fault tolerance.
Prachi et al. [27] Sanhaji et al. [28] Krishna et al. [29] Kumar et al. [30] Gul et al. [31] Gul et al. [32]	BOA & ACO LEACH Neural Network IDCNN Cluster head Selection Cluster head Selection	High alive nodes Less energy deployment Delay is reduced Loss is also reduced Energy consumption is reduced Throughput is high The energy utilization is low The life span is increased The cost is reduced The energy consumption is reduced	Should deliberate fault tolerance Need to consider the time analysis The experimental result should be considered Need to consider the Stability analysis Need to consider the Scalability analysis The quantity and accuracy of data from various CH robots may vary

**Table 2:** List of Symbols.

Symbols	Description
$E^{i}$	Initial Energy
$E_{el}$	Electronic Energy
$E_{ea}$	Data time aggregation
di	Distance
$di_{0}$	Threshold energy
$E_{pam}$	Energy of power allocation
$E_{fr}$	Requisite Energy
$E_{1}$	Energy for idle state
$E_{C}$	Energy Cost
$t_{pi}$	Predicted Value
$y_{pi}$	Actual values
$X_{mean}$	Geometric Mean

**Table 3:** Simulation Parameters.

Parameter	Value
Path construction	100 × 100 m^2^
Sink node location	(50,50)-i.e., center of the node
Count of nodes	100
Initial energy	0.5 J
Transmission energy	50 nJ
Reception energy	50 nJ
Electric energy	50 nJ
Transmit amplifier type	10 × 10^−12^
Amplifier energy	0.0013
Data aggregation energy	5 nJ
Count of rounds	2000
Data packet size	4000
PDR	0.96 to 1
Security	0 to 5

**Table 4:** Statistical Analysis on BEA-SSA compared with other models for 100 nodes.

Metrics	BEA-SSA	GWO [26]	MOFPL [22]	PRO	SSA	BES	ROA	HGS	SSO	RCSO [21]	FCR [20]
Best	0.007	0.001	0.009	0.002	0.003	0.002	0.001	0.003	0.002	0.002	0.002
Worst	0.597	0.597	0.598	0.598	0.598	0.598	0.598	0.598	0.597	0.597	0.597
Mean	0.220	0.211	0.209	0.237	0.228	0.208	0.229	0.213	0.214	0.213	0.211
Median	0.170	0.159	0.161	0.217	0.196	0.158	0.199	0.167	0.167	0.167	0.162
STD	0.187	0.193	0.195	0.194	0.193	0.196	0.192	0.192	0.192	0.192	0.194

**Table 5:** Statistical Analysis on BEA-SSA compared with other models for 200 nodes.

Metrics	BEA-SSA	GWO [26]	MOFPL [22]	PRO	SSA	BES	ROA	HGS	SSO	RCSO [21]	FCR [20]
Best	0.009	5.430	0.0003	1.349	0.002	1.780	1.950	0.001	1.610	0.001	0.007
Worst	0.598	0.597	0.597	0.597	0.597	0.597	0.598	0.598	0.598	0.598	0.598
Mean	0.223	0.223	0.213	0.212	0.213	0.214	0.217	0.215	0.219	0.214	0.214
Median	0.175	0.184	0.165	0.165	0.166	0.166	0.173	0.167	0.177	0.167	0.166
STD	0.187	0.190	0.192	0.193	0.192	0.191	0.191	0.190	0.192	0.191	0.192

## Data Availability

Not applicable.
